# Complete Bilateral Hippocampal Diffusion Restriction and Reversible Amnesia Following Opiate, Cocaine, and Benzodiazepine Abuse

**DOI:** 10.7759/cureus.12651

**Published:** 2021-01-12

**Authors:** Deborah Huang, Rimas V Lukas

**Affiliations:** 1 Neurology, Northwestern University, Feinberg School of Medicine, Chicago, USA; 2 Neuro-Oncology, Lou Malnati Brain Tumor Institute, Chicago, USA

**Keywords:** clinical neurology, magnetic resonance imaging, mri, hippocampal, diffusion restriction, cocaine, opiates, amnesia

## Abstract

The hippocampus is a crucial component of the circuits involved in memory formation and recall. Bilateral hippocampal lesions can lead to profound anterograde amnesia. As a highly vascularized structure, the hippocampus is susceptible to ischemia from hypoxic and toxic insults. Infarction of bilateral hippocampi as a result of cocaine use, while rare, is well described in the literature. Combined opiate and stimulant abuse also cause dysfunction of this structure. We present a case of complete bilateral hippocampal diffusion restriction and anterograde amnesia after heroin, cocaine, and benzodiazepine abuse, consistent with opioid-associated amnestic syndrome, as well as a remarkable resolution of amnesia months later.

## Introduction

The hippocampus is a crucial component of the limbic system that is responsible for memory encoding and retrieval [1]. It is also an area of the brain that is highly vascularized yet lies in a watershed arterial distribution, making it particularly vulnerable to ischemic injury. Insults, such as toxicity from drugs of abuse, hypoglycemia, seizures, and infectious encephalitis, have been reported leading to damage of one or both hippocampal formations [2]. These cases classically manifest as an anterograde amnesia, with varying potential for recovery based on the extent of insult [3,4].

In the emergency department (ED), differential causes of encephalopathy, a broad term meaning any diffuse disease of the brain that alters brain structure or function, include intoxication, hypoxia, seizure, infection, and metabolite derangements, sometimes in combination. More rarely, amnesia may be a part of the clinical presentation as well. Anterograde amnesia and retrograde amnesia often occur in conjunction, but as anterograde memory is more easily disrupted, it can occur in the absence of retrograde amnesia [5]. The differential for amnesia is broad as well. This ranges from structural lesions affecting the medial temporal lobe and areas around the third ventricle, sometimes as a result of traumatic brain injury (TBI), cysts, or tumors, as well as Wernicke-Korsakoff syndrome and transient disruptions of the memory circuits as seen in transient global amnesia (TGA), transient epileptic amnesia (TEA), and electroconvulsive therapy [5]. Once recognized, the presence of amnesia should prompt a close review of the differential, as multiple pathologies may lead to amnesia but differ in prognosis and treatment [2,4].

## Case presentation

A 30-year-old man with a history of polysubstance abuse, multiple suicide attempts, attention deficit hyperactivity disorder (ADHD), and recent methicillin-sensitive Staphylococcus aureus (MSSA) bacteremia presented to the ED with profound anterograde amnesia. The patient was able to recall biographical information, as well as events from more than two weeks before presentation. On chart review of outside hospital records, the patient had been admitted to the medicine service one month prior and initiated on two weeks of intravenous ceftriaxone monotherapy for uncomplicated MSSA bacteremia. He was to complete the second week of antibiotics at home via a peripherally inserted central catheter. However, the patient could not remember any events from the two weeks preceding his arrival to our ED, including how he was brought to the ED and whether he received antibiotic therapy at home.

**Figure 1:**
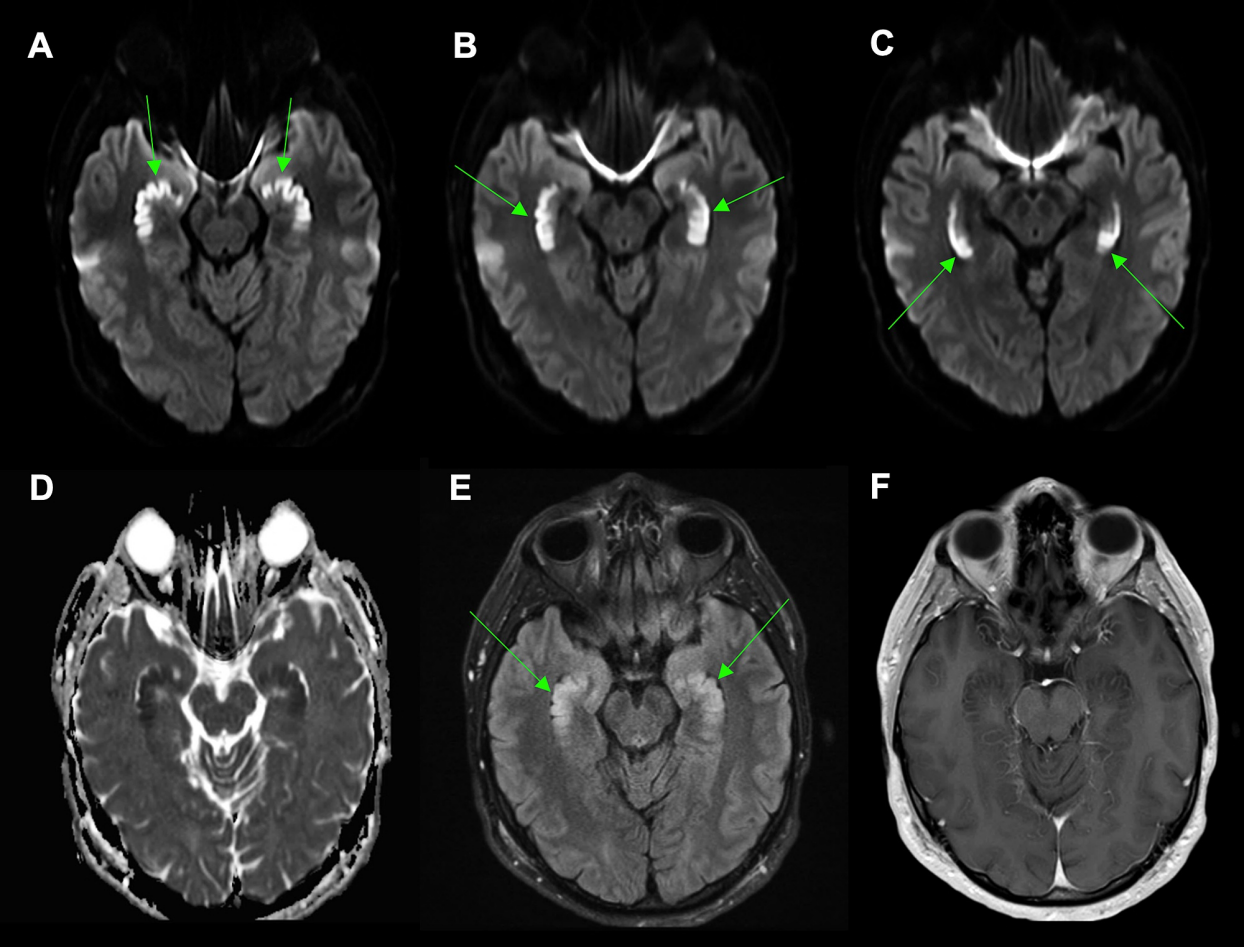
Magnetic resonance imaging of the brain. Diffusion-weighted imaging (DWI) demonstrates bilateral, symmetric hyperintensity (arrows) in the (A) heads, (B) bodies, and (C) tails of the hippocampi. (D) The apparent diffusion coefficient (ADC) correlate map shows a corresponding low signal, consistent with diffusion restriction. (E) Abnormal T2-fluid-attenuated inversion recovery (FLAIR) hyperintensity is present in the hippocampi (arrows). (F) Axial T1-weighted imaging shows no abnormal contrast enhancement.

On mental status exam, the patient recalled zero out of three items after five minutes, even when given multiple-choice options. Attention, language, executive, visuospatial functioning, calculation, and abstraction abilities remained fully intact. Physical and neurological examinations were otherwise normal. Magnetic resonance imaging (MRI) demonstrated diffusion restriction throughout the bilateral hippocampi (Figure 1). No other areas of diffusion restriction, hypo- or hyperintensities, or contrast-enhancing lesions were identified. Electroencephalogram (EEG), cerebrospinal fluid, computed tomography (CT) angiography imaging of the head and neck, and comprehensive metabolic panel were normal. Urine toxicology returned positive for cocaine, benzodiazepines, and opiates. Specific testing for fentanyl or synthetic opiates was not performed. The patient was known to have a history of heroin, cocaine, ecstasy, and alcohol abuse. He reported a diagnosis of ADHD but was never formally diagnosed with other psychiatric conditions.

The patient’s memory had not improved one month later, and arrangements were made for the patient to reside in a skilled nursing facility during substance abuse rehabilitation. Within 10 months, the patient returned to a different ED in acute alcohol withdrawal. It is unclear how he managed to leave the skilled nursing facility or resume regular alcohol consumption. A non-contrast CT scan of the brain was reportedly normal, with no mention of temporal or hippocampal atrophy. Formal neurocognitive testing and repeat MRI brain were not conducted due to the short nature of this hospital stay, but the patient no longer displayed signs of amnesia. He was able to describe multiple events from the preceding four months in moderate detail. On examination, his registration and encoding abilities were intact, and he was able to recall three out of three items after five minutes.

## Discussion

The role of the hippocampus in memory and learning was originally described by Drs. Milner and Scoville in their work with bilateral temporal lobectomy patient, H.M. [6]. Since that seminal work, multiple mechanisms have been shown to cause hippocampal lesions and result in memory impairment [3]. Bilateral hippocampal lesions portend a worse prognosis with more severe amnesia than is seen with unilateral lesions [7]. In 2017, Bhattacharyya et al. described 16 patients with bilateral hippocampal diffusion restriction, with or without basal ganglia, cerebellar, or thalamic involvement. Lesions were attributed to hypoxic, hypoxic-ischemic, ictal, or other mechanisms, such as embolic stroke and meningoencephalitis, with varying imaging findings but similar clinical presentations of anterograde amnesia amongst the group. All surviving patients in this case series remained amnestic up to 20 months later.

We describe a case of complete bilateral hippocampal diffusion restriction in a patient who presented initially with profound anterograde amnesia and substantial memory improvement after 12 months. While clinical history was limited given the patient’s memory impairment, he was able to corroborate a history of ingestion of benzodiazepines, opioids, and cocaine. With a history of alcohol intoxication and polysubstance abuse, this patient was evaluated for Wernicke-Korsakoff syndrome and given empiric thiamine supplementation. Usually a result of thiamine deficiency from chronic malnutrition, Wernicke encephalopathy is characterized by confusion, ataxia, and ocular abnormalities. About 50% to 80% of patients with Wernicke encephalopathy may develop Korsakoff syndrome, leading to a permanent anterograde amnesia with or without confabulation, retrograde amnesia, and executive deficits [8]. MRI T2-fluid-attenuated inversion recovery (FLAIR) sequences in 50% of patients may show hyperintense lesions in the fornix, medial thalamus, mammillary bodies, periventricular area around the third ventricle, and/or periaqueductal areas [9].

The differential diagnosis for this patient also includes TEA, TGA, and hippocampal stroke. These conditions lead to anterograde amnesia of variable duration [4,9]. TEA is characterized by repetitive events of short-lived anterograde amnesia and responds well to anti-seizure medication. TEA is thus a treatable condition that should remain on the differential in all amnesia evaluations. Amnesia seen with TGA lasts less than 24 hours and does not affect other cognitive functions. It is unusual for TGA to recur. In hippocampal strokes, amnesia is usually short-lived, but in a small proportion of cases, amnesia may be permanent and accompanied by cognitive symptoms such as anomia or executive difficulties [9]. Hippocampal strokes may be misdiagnosed as TGA if no MRI is obtained and symptoms improve. However, it is important to distinguish between the two, as hippocampal strokes are typically embolic and should prompt further evaluation for an embolic source. The complete bilateral hippocampal diffusion restriction seen in our patient is unlikely to represent a stroke, as each hippocampus is supplied by branches of both anterior choroidal and posterior cerebral arteries. A stroke of the entire hippocampus would require embolism to bilateral anterior and posterior circulations. The hippocampus is furthermore spared in hypotensive strokes because of its rich blood supply from multiple arteries [10].

Our patient did not present with the classic symptoms of Wernicke encephalopathy, nor did he have MRI lesions characteristic of Korsakoff syndrome. He had no evidence of seizure activity on EEG, though EEG may not reflect prior ictal events and can sometimes be normal in subcortical seizures. Follow-up imaging did not show regional hippocampal atrophy to suggest transient epileptic amnesia [9]. Due to the patient’s recent MSSA bacteremia, embolic stroke to the posterior circulation was considered a potential etiology but felt to be unlikely. Embolic and infectious workups were negative.

Hippocampal lesions from anoxic injury portend a poorer prognosis [2,11]. Hypoxia has been shown in rat models to lead to hyperpolarization and neuronal injury in the hippocampal CA1 region [12]. There are multiple entities known to cause hippocampal ischemia and hypoxia, with varying prognoses [4]. Ischemia after cardiac arrest, for example, carries a low likelihood of memory recovery [2]. Cocaine-induced vasospasm and carbon monoxide poisoning are rare but well-recognized causes of bilateral hippocampal ischemia [13,14]. Case reports and a few case series in the literature suggest that patients with hypoxia but no evidence of ischemia, and those with histotoxic hypoxia such as that seen with carbon monoxide poisoning, may be more likely to recover regardless of initial imaging severity [4].

The Massachusetts Department of Public Health reported a cluster of unusual amnestic cases between 2012 and 2016 in young patients [15]. Thirteen out of 14 patients reported a history of substance use, with 12 specifically reporting opiate use. Eight patients tested positive for opiates, two for cocaine, and two for benzodiazepines. They were found to have bilateral hippocampal restricted diffusion on MRI similar to our patient, with or without extra-hippocampal involvement, and had no other explanations for hippocampal lesions. Follow-up information was not available for the majority of these cases, but in the few patients with data available, only one had resolution of short-term memory loss. After the Massachusetts case series was described in 2017, more cases have been published to support the establishment of a new opioid-induced amnestic syndrome, sometimes in patients with concurrent stimulant or benzodiazepine abuse [16]. Benzodiazepine use in these patients is likely incidental, as benzodiazepines are not known to cause hippocampal neurotoxicity. However, benzodiazepine use may contribute to respiratory depression. Barash et al. recently established a formal definition for this opioid-associated amnestic syndrome (OAS), defined as acute onset memory loss lasting more than 24 hours and bilateral hippocampal signal abnormalities on brain imaging in the setting of opioid use. They also validated a set of diagnostic criteria for “confirmed,” “probable,” and “possible” OAS, based on 40 unique cases of bilateral hippocampal diffusion restriction identified within the literature [17]. All cases consist of a new onset amnesia lasting at least 24 hours. “Possible” OAS additionally includes either positive opioid toxicology, known history of opioid use, or bilateral hippocampal injury on CT or MRI. “Probable” OAS cases have the bilateral hippocampal imaging findings and a known history of opioid use. OAS is “confirmed” when toxicology verifies opioid use. Of the 40 patients identified, there were 20 confirmed, 10 probable, and 10 possible OAS cases. Our patient meets all diagnostic criteria for “confirmed OAS” [17].

Animal models and human case reports demonstrate how exquisitely sensitive the hippocampus is to systemic changes in perfusion, oxygenation, and exogenous agents that affect its metabolism and cellular activity. Hippocampal injury, even if temporary, disrupts memory encoding and retrieval. In one study of mice that were intubated to prevent hypoxia, fentanyl administration led to hippocampal and associated limbic damage [18]. μ-opioid receptors within the hippocampus, when activated by exogenous opioids, inhibit gamma-aminobutyric acid (GABA)-ergic interneurons and prevent appropriate modulation of hippocampal neuronal activity, leading to hypermetabolism and excitotoxic damage. Reactive oxygen species as byproducts of hypermetabolism may induce apoptosis and cause additional neurotoxic effects [16]. Additionally, OAS patients might sustain varying degrees of hypoxemia as a result of opioid-induced respiratory depression, compounding the damage from increased metabolic demands on vulnerable hippocampi [19]. As all of the reported OAS patients came to medical attention following out-of-hospital events, this last hypothesis is difficult to confirm but plausible. The high sensitivity of hippocampal neurons to ischemia may explain why OAS patients display hippocampal diffusion restriction on MRI without significant cortical findings. 

Our patient was fortunate to have recovered from his amnesia over the course of several months, but many patients with bilateral hippocampal injury never return to their prior cognitive abilities [15]. It is not immediately clear why his memory returned. With no evidence of structural abnormalities in either hippocampus on his initial or follow-up brain imaging, this patient was likely spared from permanent tissue ischemia.

The hippocampus is the only known area of the human brain where physiologic neurogenesis continues even in adulthood [20]. Could there have been repair or regeneration of injured hippocampal neurons? If so, should we expect a higher number of recoveries from OAS? Research about post-traumatic amnesia recovery after TBI is ongoing, but at this time OAS remains an uncommon entity. Long-term studies would be informative but difficult to conduct. It is largely unknown what patient characteristics or outcomes predict amnesia recovery in cases such as ours. Further research is warranted to better understand OAS pathophysiology, the role of polysubstance use in its development, and why some patients appear to recover more completely than others from this unique amnestic syndrome.

## Conclusions

The hippocampus is an area of the brain that is critical for memory formation and particularly vulnerable to hypoxia, hypermetabolic demands, and toxic insults. This vulnerability may be associated with a severe anterograde amnestic syndrome at onset and varying potential for recovery. We present a case of OAS, an emerging amnestic syndrome, as well as a differential to consider when evaluating amnestic patients. It is important to recognize the different etiologies of bilateral hippocampal injury and amnesia to ensure that patients receive appropriate evaluation which, in turn, allows for targeted treatment and care planning.
